# Supplementary material for: Human prestige psychology can promote adaptive inequality in social influence
Source: Nat Commun. 2026 Feb 3;17:947. doi: 10.1038/s41467-026-68410-7 (PMC12868758; doi:10.1038/s41467-026-68410-7)
Supplement: Supplementary file 1 — Supplementary Information [file 41467_2026_68410_MOESM1_ESM.pdf]

Human Prestige Psychology Promotes Adaptive Inequality in Social Influence

~ Supplementary Information ~

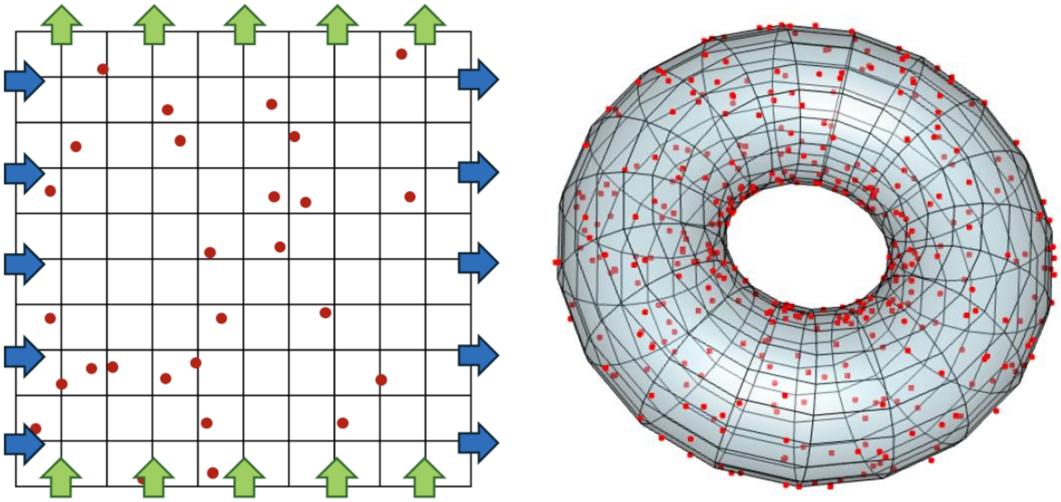

Fig. S1. Visualizations of the space in which agents exist in model 1. The left shows a 2D visualization, where agents are the red dots and the grid is the space. The arrows indicate that the sides “wrap around”, that is agents near the left side are close in the space to agents on the right side, etc. Such “wrapping around” avoids the space having edges. The right shows a 3D visualization of this space as the surface of a torus. That is, linking the left and right side of the space folds it into a tube, and then join the top and bottom folds it into a torus. However, note that while a torus might be a useful visualization, the space agents are placed in does not obey many properties of real toruses. For instance, unlike a real torus, the outer circumference is not larger that the inner circumference, and in fact they are the same length.

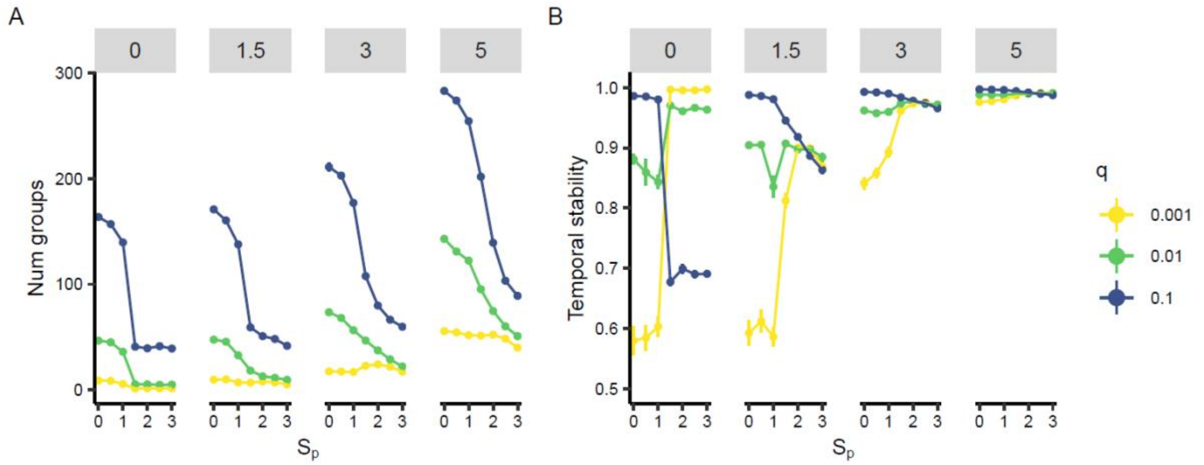

Fig S2. Effects of prestige sensitivity, s_p, distance penalty (from 0 to 5 as indicated above plots), and mutation rate, q, on cultural dynamics. All plots display the mean and standard error across 12 repeats of the model. The prestige decay rate, p, was set to 0.2. A) The number of groups in the population with more than 1 adherent. B) Temporal stability. The Gini plot and spearman's correlation plots are excluded as they are given in main text Figure 1.

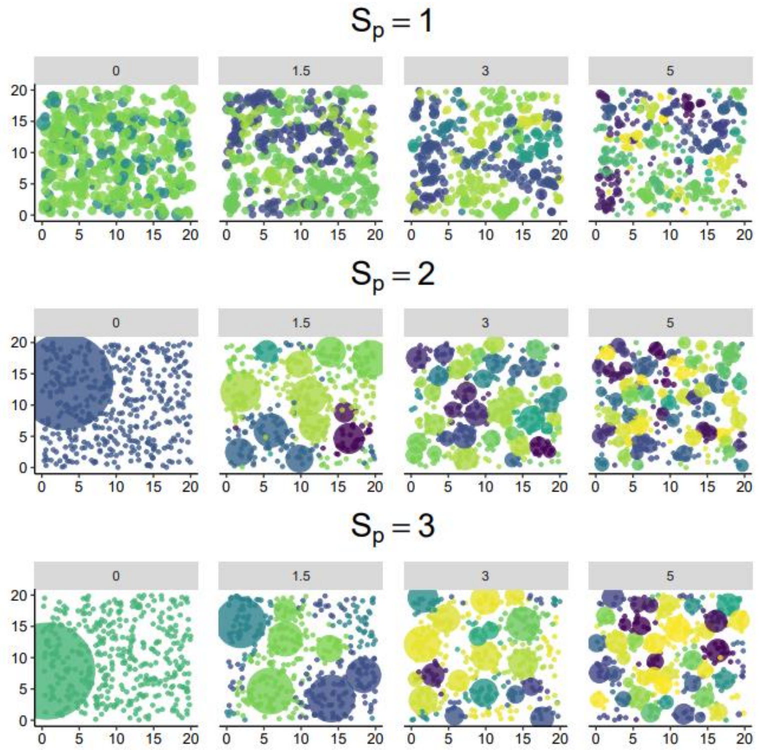

Fig S3. Bubble plot showing the x coordinates (x axis), y coordinates (y axis), belief (colour) and relative prestige (size of point) of each individual in a single run. Panels split between values of distance penalty. The prestige exponent (s_p) is set to 1 (top row), 3 (middle row), or 5 (bottom row). For each plot, the innovation rate (q) was set to 0.001 p to 0.2.

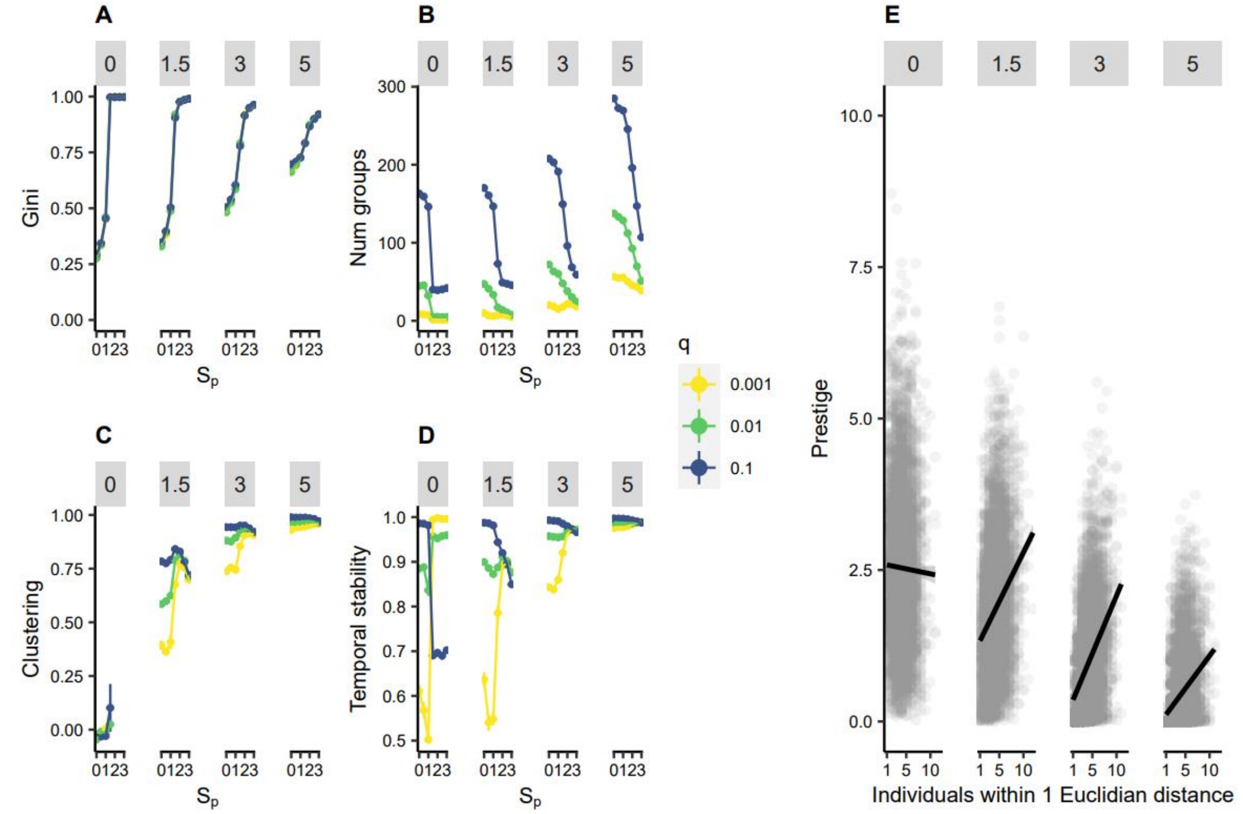

Fig S4. Results where the prestige decay rate, p, is set to 0.5. All plots display the mean and standard error across 12 repeats of the model for p = 0.5. Sub-panels vary the distance penalty (d) and colored lines vary the innovation rate (q). **A)** The Gini coefficient. As before, s_p has much greater impact than does d; R^2 = 0.58 and 0.01, respectively. **B)** The number of groups in the population with more than 1 adherent. **C)** The clustering statistic. Values beyond s = 2 when d = 0 are not shown because the population is entirely homogenous and so the statistic cannot be computed. **D)** Temporal stability. **E)** Spearman's correlations between the number of individuals within 1 Euclidian distance and an individual's prestige. Points show mean correlations across 12 model repeats and lines show linear regression. Correlation coefficients are: -.026, .263, .426. .424.

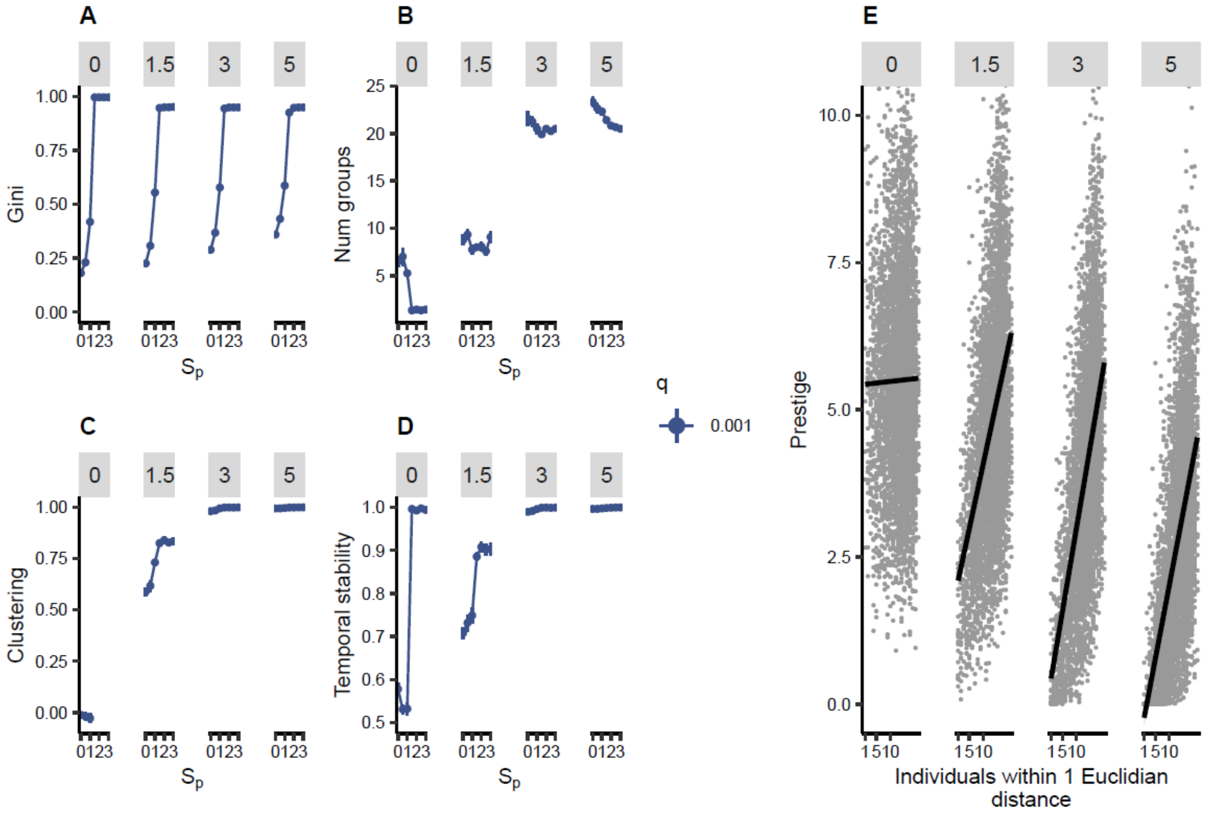

Fig S5. Results where individuals are placed into evenly spaced and equally sized clusters, or "villages". All plots display the mean and standard error across 12 repeats of the model for p = 0.5. To save on computation time, the innovation rate (q) was held constant at 0.001. The results are qualitatively consistent with those where individuals are located randomly on the grid. However, the assumption of pre-clustered villages increases the overall clustering of beliefs as villages typically adopt the same fixed belief.

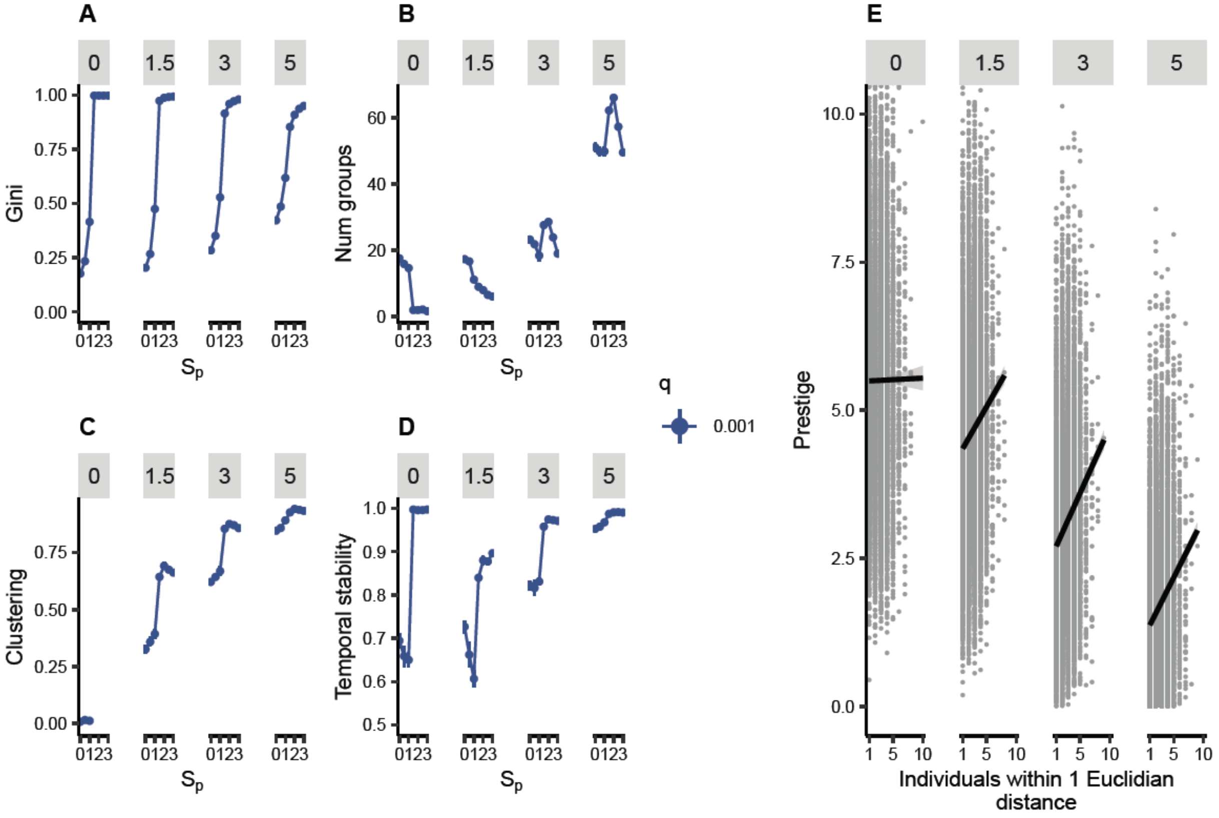

Fig S6. Results where the population size is increased to 800, but the size of the grid is kept at 20x20. All plots display the mean and standard error across 12 repeats of the model for p = 0.5. To save on computation time, the innovation rate (q) was held constant at 0.001. The results are qualitatively consistent with those presented in the main text, though the larger population size slightly decreases the clustering of beliefs and increases temporal stability.

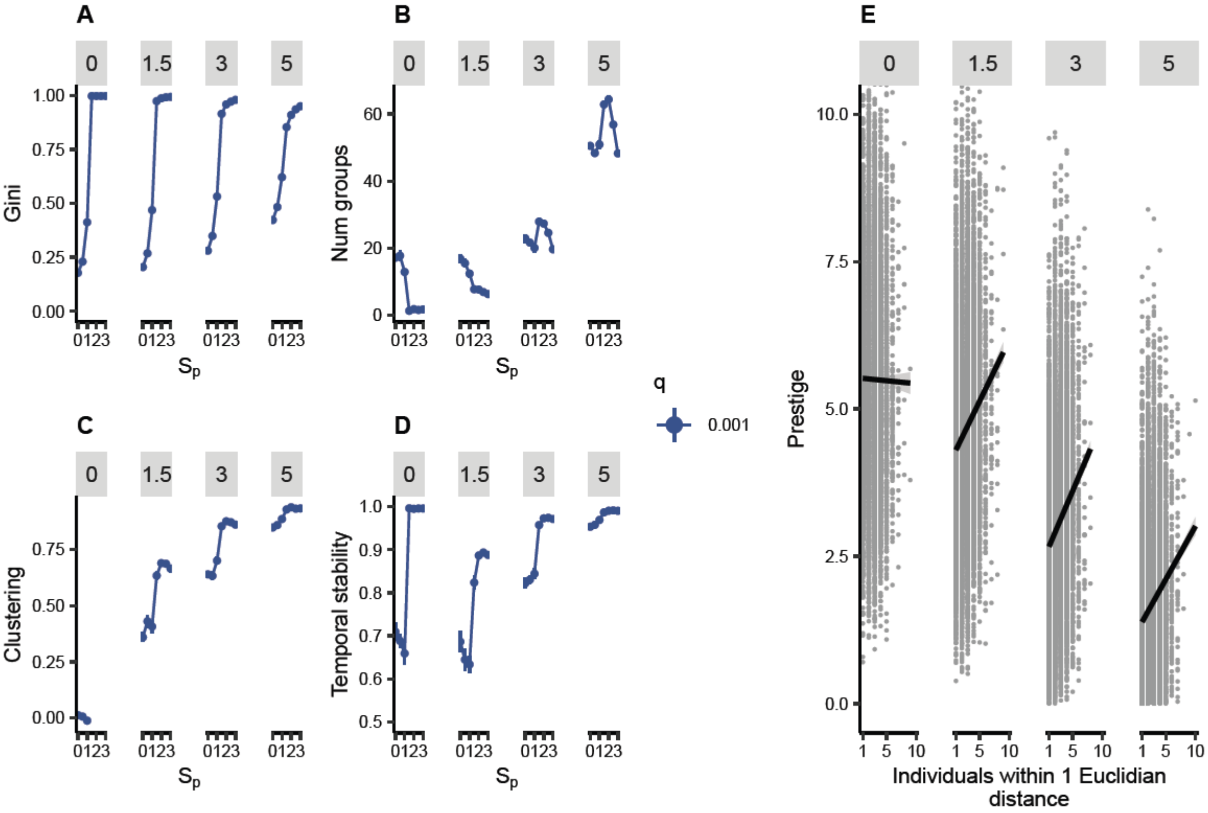

Fig S7. Results where the population size is increased to 800 and the size of the grid is increased to 40 x 40. All plots display the mean and standard error across 12 repeats of the model for p = 0.5. To save on computation time, the innovation rate (q) was held constant at 0.001. The results are qualitatively consistent with those presented in the main text, though the larger yet more diffuse population size slightly decreases the clustering of beliefs and increases temporal stability.

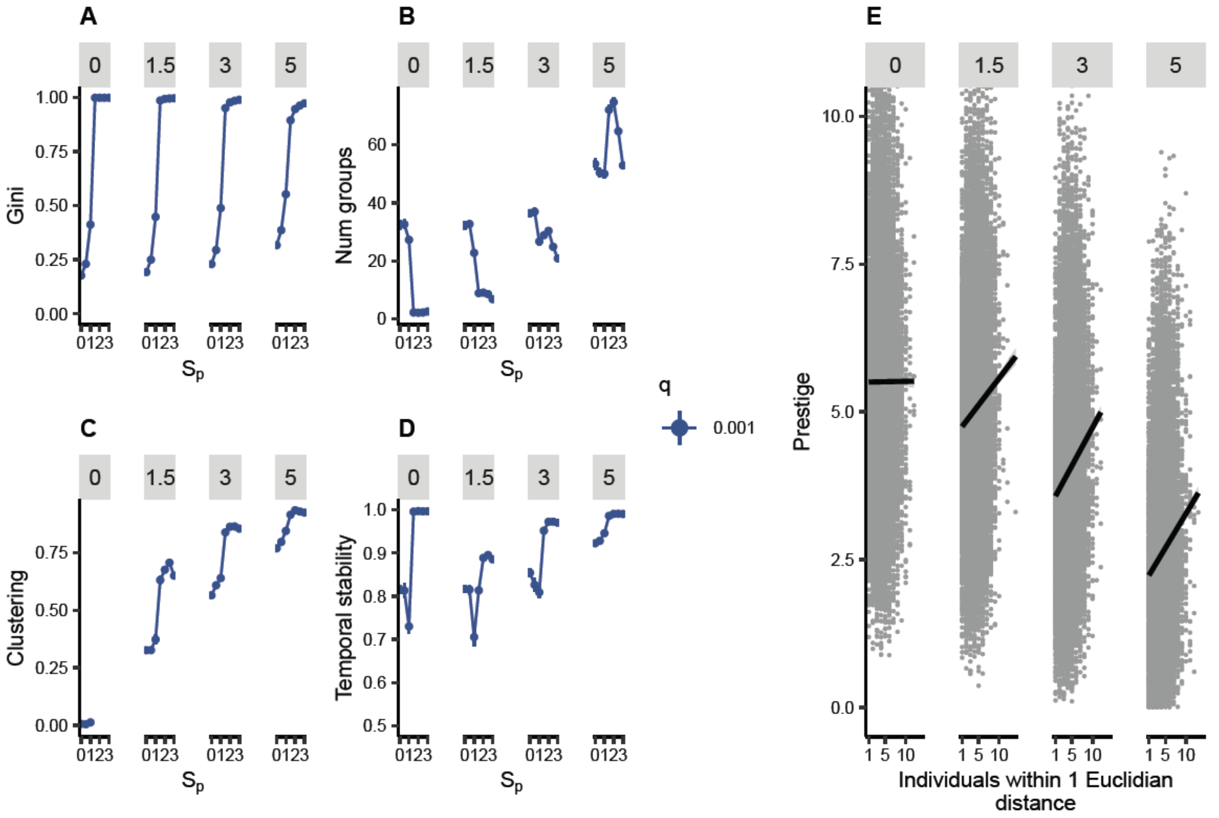

Fig S8. Results where the population size is increased to 1600 and the size of the grid is increased to 40 x 40. All plots display the mean and standard error across 12 repeats of the model for p = 0.5. To save on computation time, the innovation rate (q) was held constant at 0.001. The results are qualitatively consistent with those presented in the main text, though the larger population size slightly decreases the clustering of beliefs and increases temporal stability.

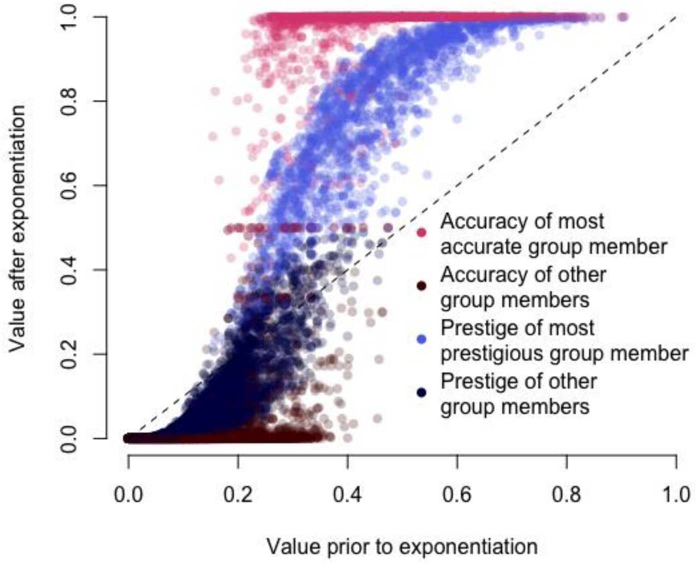

Fig S9. The impact of prestige and accuracy sensitivity on the social influence of group mates. Each point shows the normalized value of a group member's accuracy (red/pink) and prestige (dark/light blue), before and after exponentiation, from 2000 randomly selected trials, using median posterior samples as point estimates for the two sensitivity parameters. While the top scoring group member always has a disproportionate amount of influence (i.e. is above the black dashed line), this is particularly extreme with accuracy where the top scoring group member typically has *all* the influence. In contrast, the most prestigious group member exerts disproportionate influence there is a smoother gradation between participants.

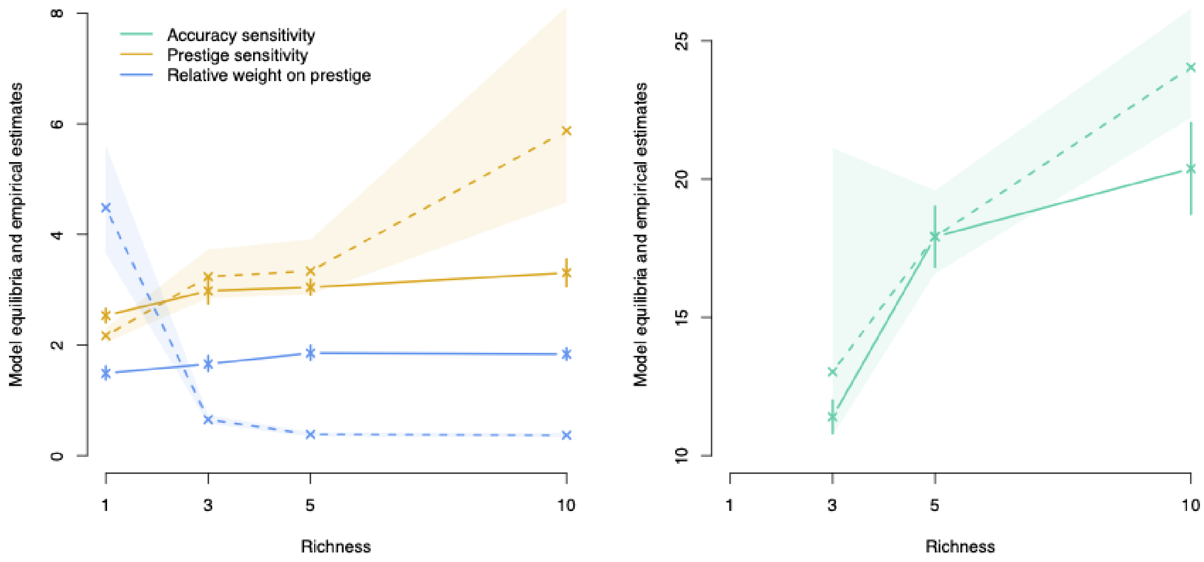

Fig S10. Comparison of empirical results (dashed lines with shaded regions indicating the 95% credible interval) and predictions of the evolutionary model (solid lines with error bars indicating 2 standard errors) across information richness conditions. The model and data are in near perfect agreement regarding the sensitivity parameters, but diverge regarding the relative weight of prestige and accuracy. Theoretical results are the mean-of-means allele values after 5000 generations. Note the y-axis is linear and results are split across two panels as the range of accuracy sensitivity does not overlap those of the other two parameters.

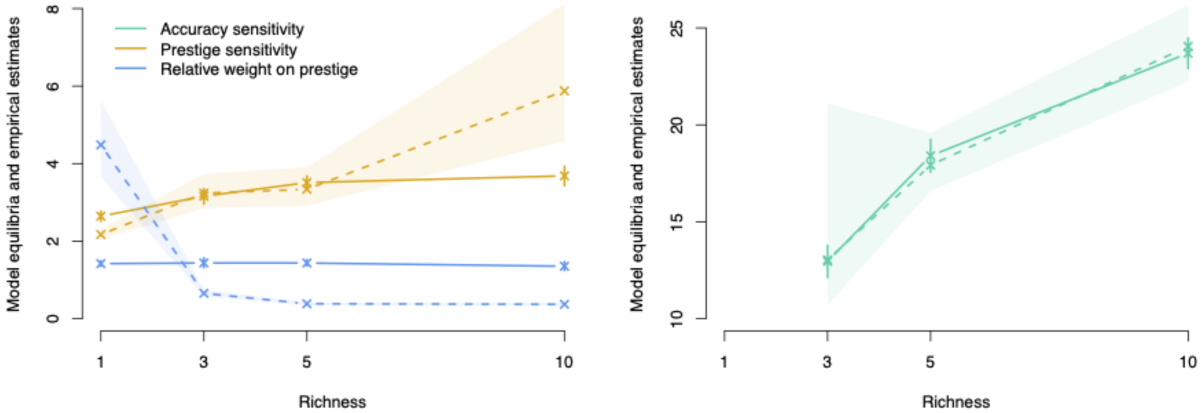

Fig. S11. Comparison of empirical results (dashed lines with shaded regions indicating the 95% credible interval) and predictions of the evolutionary model (solid lines with error bars indicating 2 standard errors) across information richness conditions. In this case, the distribution from which individuals' skill at the task is drawn is changed from a Beta(4, 4) distribution, to a Beta(1, 1) distribution. This is a flat distribution between 0 and 1 and so it increases the variance in skill in the population. Despite this modification, the qualitative results are unchanged: as in the main paper the model and data are in near perfect agreement regarding the sensitivity parameters, but diverge regarding the relative weight of prestige and accuracy. Theoretical results are the mean-of-means allele values after 5000 generations. Note the y-axis is linear and results are split across two panels as the range of accuracy sensitivity does not overlap those of the other two parameters.

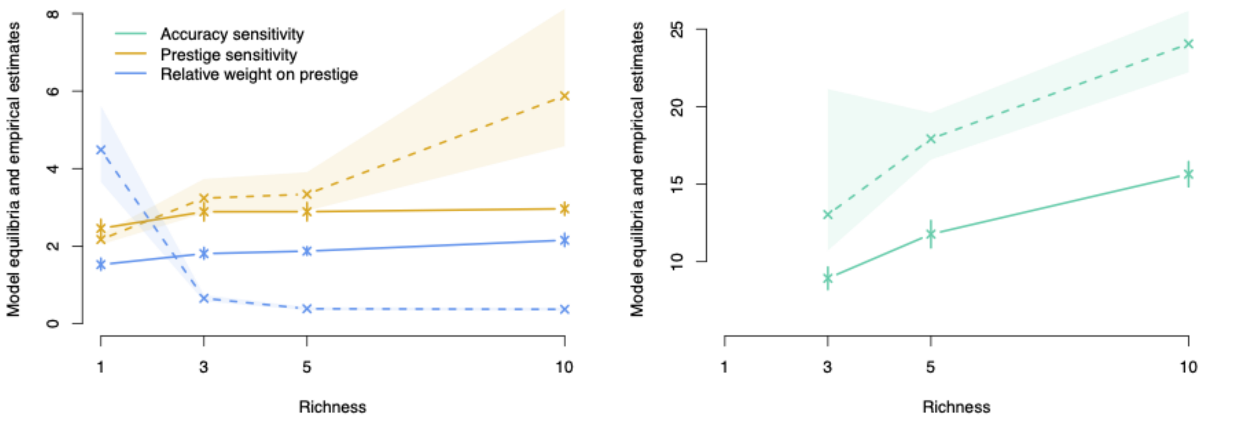

Fig. S12. Comparison of empirical results (dashed lines with shaded regions indicating the 95% credible interval) and predictions of the evolutionary model (solid lines with error bars indicating 2 standard errors) across information richness conditions. In this case, the distribution from which individuals' skill at the task is drawn is changed from a Beta(4, 4) distribution, to a Beta(10, 10) distribution. This is a relatively dense distribution, centered on 0.5, and it decreases the variance in skill in the population. Despite this modification, the broad results are unchanged: prestige sensitivity is strong enough to produce unequal prestige distributions, accuracy sensitivity is much higher, and the model diverges from the experimental results regarding the relative weights of prestige and accuracy. Nonetheless, there are minor differences between this model and that in the main paper, principally accuracy sensitivity is decreased somewhat. In addition, the theoretical relationship between richness and prestige sensitivity is flatter, but it is still positive. These differences can be interpreted as the lower variance in the population somewhat undermining the need for prestige-based or accuracy-based copying in the first place. Given that all individuals are relatively similar in their ability at the task, there is less need to be discriminating in terms of who to copy. Theoretical results are the mean-of-means allele values after 5000 generations. Note the y-axis is linear and results are split across two panels as the range of accuracy sensitivity does not overlap those of the other two parameters.

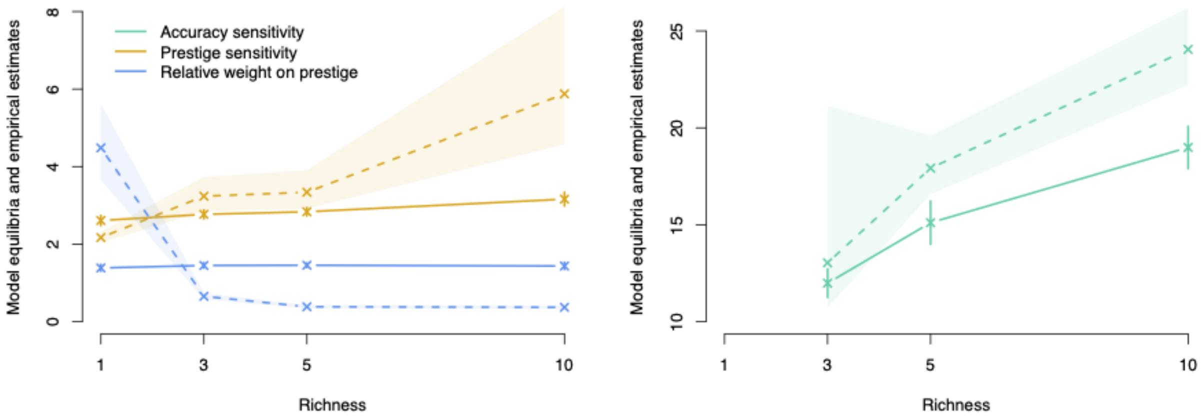

Fig. S13. Comparison of empirical results (dashed lines with shaded regions indicating the 95% credible interval) and predictions of the evolutionary model (solid lines with error bars indicating 2 standard errors) across information richness conditions. In this case, the size of groups formed by individuals is decreased from 20 to 10. Despite this modification, the broad results are unchanged: prestige sensitivity is strong enough to produce unequal prestige distributions, accuracy sensitivity is much higher than prestige sensitivity, and the model diverges from the experimental results regarding the relative weights of prestige and accuracy. Nonetheless, there are minor differences between this model and that in the main paper, specifically accuracy sensitivity is decreased a little and the theoretical relationship between richness and prestige sensitivity appears flatter, but it is still positive. Theoretical results are the mean-of-means allele values after 5000 generations. Note the y-axis is linear and results are split across two panels as the range of accuracy sensitivity does not overlap those of the other two parameters.

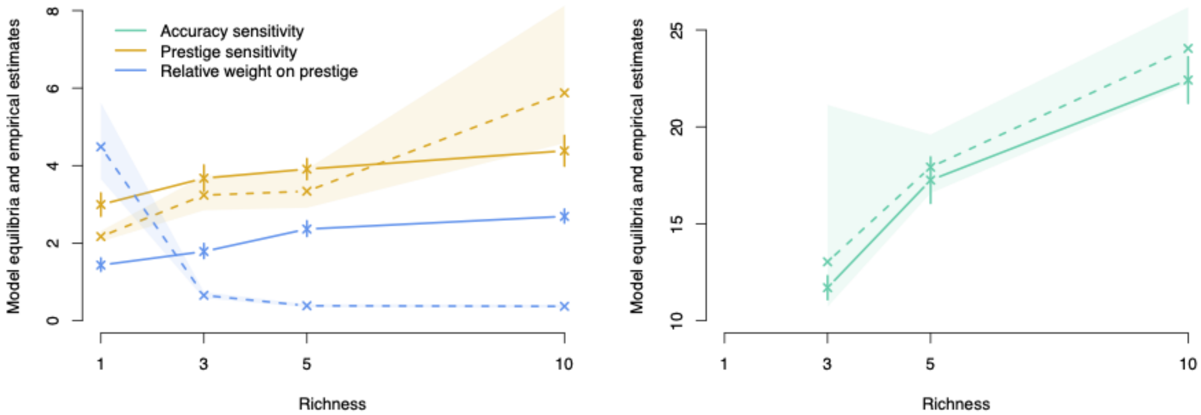

Fig. S14. Comparison of empirical results (dashed lines with shaded regions indicating the 95% credible interval) and predictions of the evolutionary model (solid lines with error bars indicating 2 standard errors) across information richness conditions. In this case, the size of groups formed by individuals is increased from 20 to 50. Despite this modification, the qualitative results are unchanged: as in the main paper the model and data are in near perfect agreement regarding the sensitivity parameters, but diverge regarding the relative weight of prestige and accuracy. Theoretical results are the mean-of-means allele values after 5000 generations. Note the y-axis is linear and results are split across two panels as the range of accuracy sensitivity does not overlap those of the other two parameters.

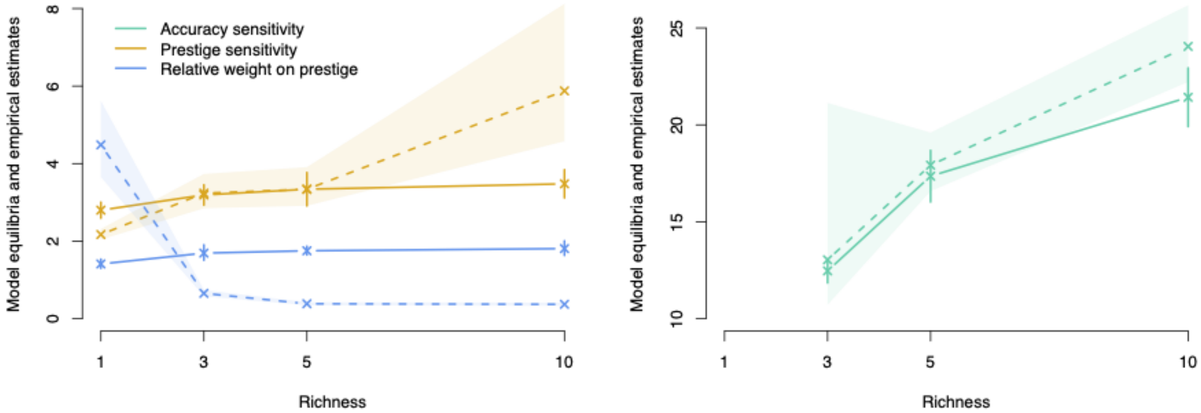

Fig. S15. Comparison of empirical results (dashed lines with shaded regions indicating the 95% credible interval) and predictions of the evolutionary model (solid lines with error bars indicating 2 standard errors) across information richness conditions. In this case, the total population size is reduced from 2000 to 1000. Despite this modification, the qualitative results are unchanged: as in the main paper the model and data are in near perfect agreement regarding the sensitivity parameters, but diverge regarding the relative weight of prestige and accuracy. Theoretical results are the mean-of-means allele values after 5000 generations. Note the y-axis is linear and results are split across two panels as the range of accuracy sensitivity does not overlap those of the other two parameters.

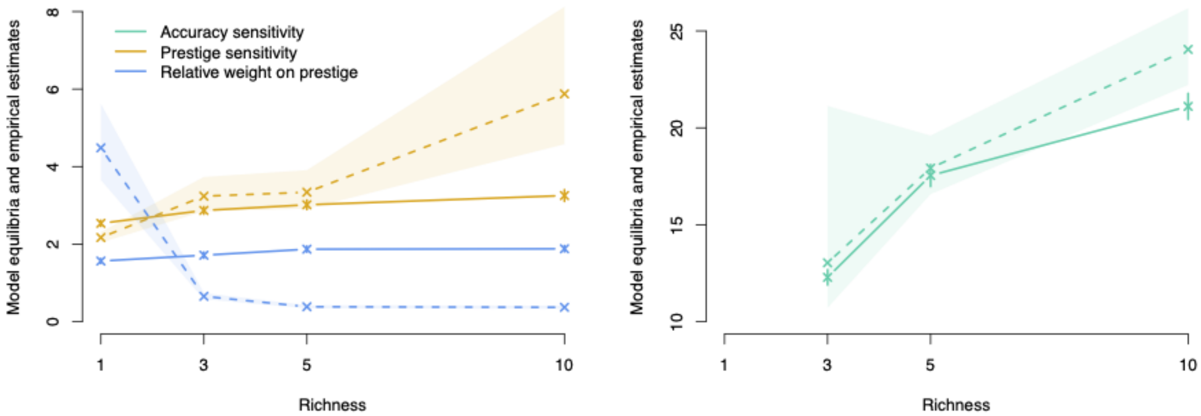

Fig. S16. Comparison of empirical results (dashed lines with shaded regions indicating the 95% credible interval) and predictions of the evolutionary model (solid lines with error bars indicating 2 standard errors) across information richness conditions. In this case, the total population size is increased from 2000 to 5000. Despite this modification, the qualitative results are unchanged: as in the main paper the model and data are in near perfect agreement regarding the sensitivity parameters, but diverge regarding the relative weight of prestige and accuracy. Theoretical results are the mean-of-means allele values after 5000 generations. Note the y-axis is linear and results are split across two panels as the range of accuracy sensitivity does not overlap those of the other two parameters.

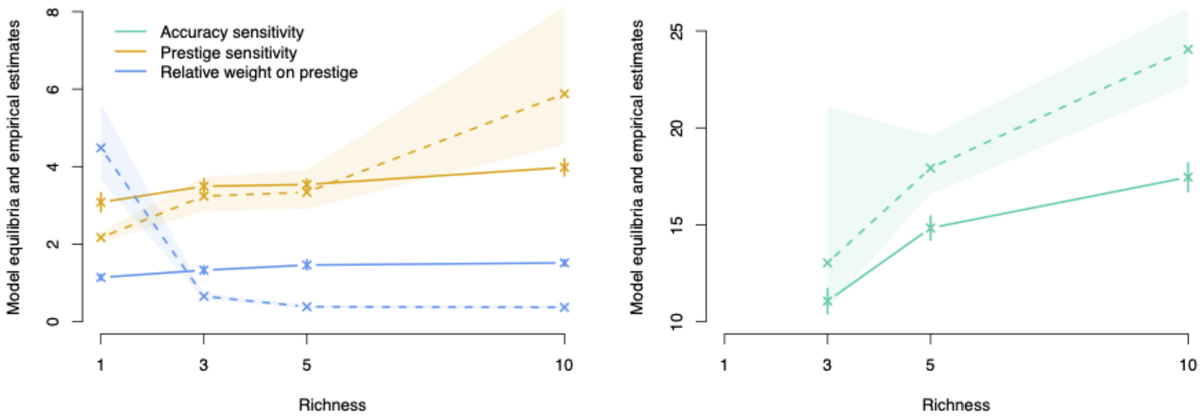

Fig. S17. Comparison of empirical results (dashed lines with shaded regions indicating the 95% credible interval) and predictions of the evolutionary model (solid lines with error bars indicating 2 standard errors) across information richness conditions. In this case, the number of trials groups complete is decreased from 40 to 20. Despite this modification, the broad results are unchanged: prestige sensitivity is strong enough to produce unequal prestige distributions, accuracy sensitivity is much higher than prestige sensitivity, and the model diverges from the experimental results regarding the relative weights of prestige and accuracy. Nonetheless, there are minor differences between this model and that in the main paper, specifically accuracy sensitivity is decreased a little and the theoretical relationship between richness and prestige sensitivity appears flatter, but it is still positive. Theoretical results are the mean-of-means allele values after 5000 generations. Note the y-axis is linear and results are split across two panels as the range of accuracy sensitivity does not overlap those of the other two parameters.

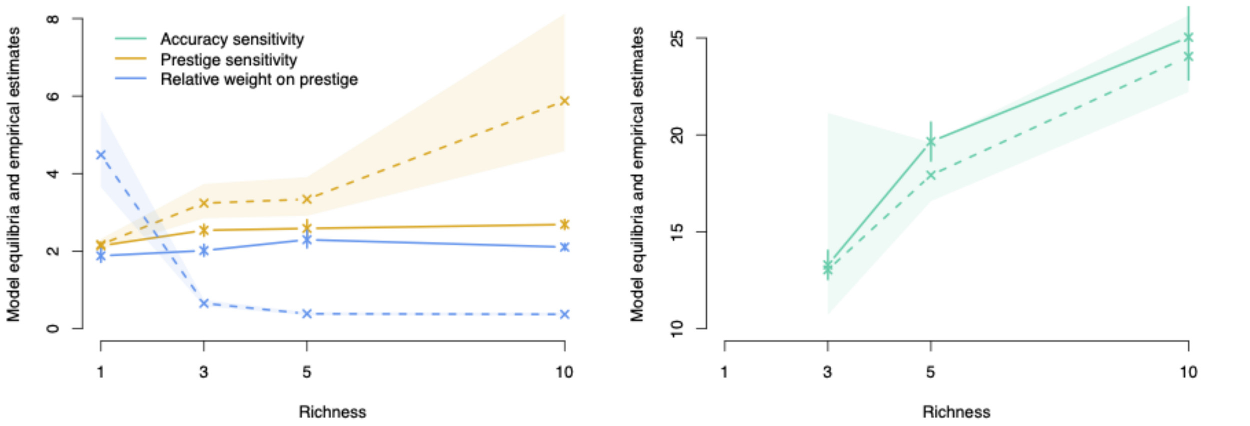

Fig. S18. Comparison of empirical results (dashed lines with shaded regions indicating the 95% credible interval) and predictions of the evolutionary model (solid lines with error bars indicating 2 standard errors) across information richness conditions. In this case, the number of trials that groups complete is increased from 40 to 80. Despite this change, the qualitative results are unchanged: as in the main paper the model and data are in near perfect agreement regarding the sensitivity parameters, but diverge regarding the relative weight of prestige and accuracy. Theoretical results are the mean-of-means allele values after 5000 generations. Note the y-axis is linear and results are split across two panels as the range of accuracy sensitivity does not overlap those of the other two parameters.

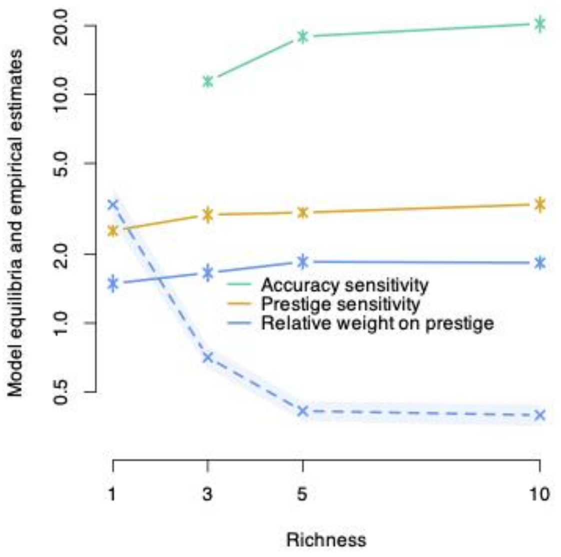

Fig S19. A reproduction of figure 3 from the main manuscript using altered priors as a sensitivity check. The revised priors are $\beta_{P,1:4} + \beta_{A,1:4} \sim \text{Exponential}(0.1)$, $s_P \sim \text{Exponential}(0.1)$, $s_A \sim \text{Exponential}(0.1)$ and $\sigma \sim \text{Exponential}(1)$. As the prior for the weight of prestige relative to accuracy was already maximally diffuse it was not altered: $\frac{\beta_{P,1:4}}{\beta_{P,1:4}+\beta_{A,1:4}} \sim \text{Beta}(1,1)$. The figure shows a comparison of empirical results (dashed lines with shaded regions indicating the 95% credible interval) and predictions of the evolutionary model (solid lines with error bars indicating 2 standard errors) across information richness conditions. The comparison is only shown for the relative weight participants put on prestige (versus accuracy) as the replicated analysis did not allow the sensitivity parameters to vary by information richness condition. As in the main analysis the model and data diverge regarding the relative weight of prestige and accuracy, thus the results are not sensitive to the priors used.

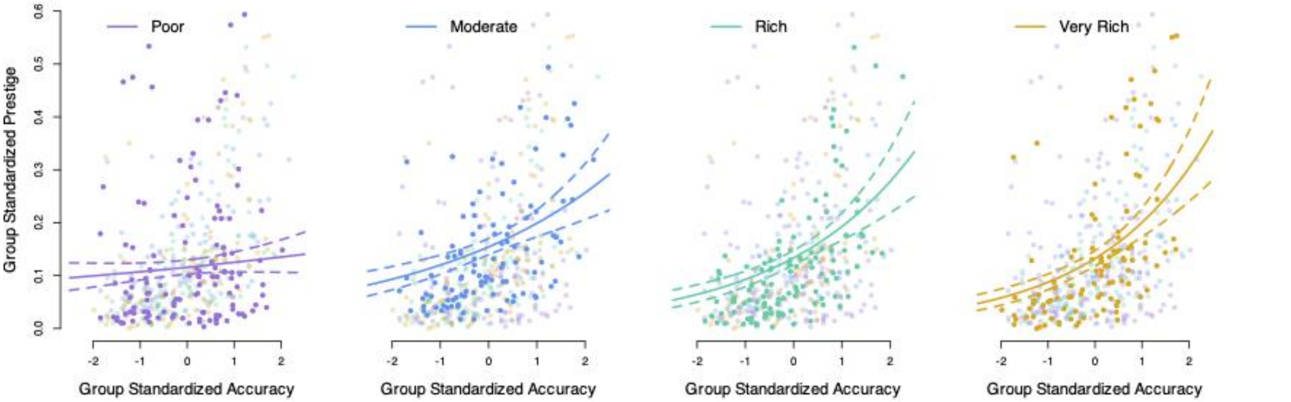

Fig S20. The relationship between asocial accuracy and prestige across the four conditions with more diffuse priors as a sensitivity check. The priors were $\beta_{3,1:4} \sim Normal(0, 10)$, $\beta_{4,1:4} \sim Normal(0, 5)$ and σ ~ Exponential(0.2). Dots are individual participants, coloured by condition, with data from the focal condition emphasized. Lines depict model estimates (median and 95% central credible intervals). The relationship is clearly positive in all conditions other than the poor condition. Moreover, the results match those produced in the main analysis, implying the results are robust to changes in the priors.

Table S1. Parameter estimates from the second analysis, presented as the median of the posterior and the 95% central credible interval. Note that, for the R^2 values, rather than a single point estimate, Bayesian methods produce a posterior distribution for the explanatory power of the model. The negative lower bound in condition 1 indicates the model's explanatory power is plausibly worse than a model without accuracy.

Parameter	Interpretation	Condition			
		Poor	Moderate	Rich	Very rich
β_3	log-prestige assuming average accuracy	-2.16 [-2.28, -2.05]	-1.87 [-1.98, -1.77]	-2.01 [-2.12, -1.91]	-2.02 [-2.14, -1.91]
β_4	Increase in prestige per sd of accuracy (log scale)	0.08 [-0.02, 0.17]	0.25 [0.15, 0.35]	0.36 [0.25, 0.46]	0.41 [0.30, 0.52]
σ	Standard deviation of prestige given accuracy	0.108 [0.098, 0.119]			
R^2	Explanatory power of model	0.002 [-0.02, 0.01]	0.23 [0.15, 0.28]	0.34 [0.28, 0.44]	0.31 [0.26, 0.36]

Table S2. Parameter estimates from the follow-up analysis that allowed the sensitivity parameters to vary by experimental condition, presented as the median of the posterior and the 95% central credible interval. Note that $s_A[1]$ has no meaning as in the poor condition accuracy values are all 0 or 1 so raising them to a power has no effect, hence the extremely wide posterior for this parameter.

Parameter	Median [95% CI]
$s_P[1]$	2.17 [2.04 2.31]
$s_P[2]$	3.24 [2.85 3.73]
$s_P[3]$	3.34 [2.91 3.91]
$s_P[4]$	5.88 [4.58 8.12]
$s_A[1]$	2.72 [0.10 14.66]
$s_A[2]$	13.04 [10.72 21.13]
$s_A[3]$	17.92 [16.58 19.60]
$s_A[4]$	24.05 [22.22 26.17]
σ_{ppt}	1.68 [1.58 1.78]
w_P [1]	28.92 [20.64 40.36]
w_P [2]	8.45 [5.75 12.42]
w_P [3]	15.06 [11.12 20.18]
w_P [4]	10.47 [7.56 14.45]
$w_A[1]$	6.44 [4.30 9.54]
$w_A[2]$	12.91 [8.83 18.90]
$w_A[3]$	39.20 [29.12 52.08]
$w_A[4]$	28.30 [20.53 38.67]

Table S3. Parameter estimates (median and 95% central credible interval) from the categorical analysis with more diffuse priors as a sensitivity check. The revised priors are $\beta_{P,1:4} + \beta_{A,1:4} \sim \text{Exponential}(0.1)$, $s_P \sim \text{Exponential}(0.1)$, $s_A \sim \text{Exponential}(0.1)$ and $\sigma \sim \text{Exponential}(1)$. As the prior for the weight of prestige relative to accuracy was already maximally diffuse it was not altered: $\frac{\beta_{P,1:4}}{\beta_{P,1:4}+\beta_{A,1:4}} \sim \text{Beta}(1,1)$. Note that while the parameter estimates for β_P and β_A are different from the analysis in the main manuscript, their ratio, $\frac{\beta_P}{\beta_A}$, which is what matters for interpreting the results, is virtually unchanged. Thus, the results are not meaningfully sensitive to the priors.

Parameter	Interpretation	Condition			
		Poor	Moderate	Rich	Very rich
β_P	Weight of prestige relative to randomness	54.2 [33.9, 86.1]	14.9 [9.2, 24.6]	37.9 [25.1, 56.4]	29.6 [19.2, 45.4]
β_A	Weight of accuracy relative to randomness	16.5 [9.8, 27.1]	21.0 [13.2, 34.6]	92.0 [61.0, 136.0]	74.7 [49.0, 114.1]
$\frac{\beta_P}{\beta_A}$	Weight of prestige relative to accuracy	3.29 [2.77, 3.97]	0.61 [0.63, 0.79]	0.41 [0.37, 0.45]	0.40 [0.35, 0.44]
s_P	Prestige sensitivity	2.71 [2.58, 2.86]			
s_A	Accuracy sensitivity	20.78 [20.48, 23.43]			
σ	Standard deviation of individual variation	1.80 [1.70, 1.92]			

Table S4. Parameter estimates from the second analysis, presented as the median of the posterior and the 95% central credible interval, with more diffuse priors as a sensitivity check. The priors were $\beta_{3,1:4} \sim Normal(0, 10)$, $\beta_{4,1:4} \sim Normal(0, 5)$ and $\sigma \sim$ Exponential(0.2). In all cases the numerical estimates are effectively unchanged indicating the model results are robust to changes in the priors. Note that, for the R^2 values, rather than a single point estimate, Bayesian methods produce a posterior distribution for the explanatory power of the model. The negative lower bound in condition 1 indicates the model's explanatory power is plausibly worse than a model without accuracy.

Parameter	Interpretation	Condition			
		Poor	Moderate	Rich	Very rich
β_3	log-prestige assuming average accuracy	-2.16 [-2.28, -2.05]	-1.87 [-1.97, -1.77]	-2.02 [-2.12, -1.92]	-2.03 [-2.14, -1.91]
β_4	Increase in prestige per sd of accuracy (log scale)	0.08 [-0.02, 0.18]	0.25 [0.15, 0.35]	0.36 [0.25, 0.47]	0.42 [0.30, 0.52]
σ	Standard deviation of prestige given accuracy	0.107 [0.098, 0.118]			
R^2	Explanatory power of model	0.002 [-0.02, 0.01]	0.23 [0.14, 0.27]	0.37 [0.27, 0.44]	0.31 [0.25, 0.35]
