# Supplementary material for: Human prestige psychology can promote adaptive inequality in social influence
Source: Nat Commun. 2026 Feb 3;17:947. doi: 10.1038/s41467-026-68410-7 (PMC12868758; doi:10.1038/s41467-026-68410-7)
Supplement: Supplementary file 2 — Reporting Summary [file 41467_2026_68410_MOESM2_ESM.pdf]

nature portfolio

Corresponding author(s): Thomas Morgan

Last updated by author(s): 15th April 2025

# Reporting Summary

Nature Portfolio wishes to improve the reproducibility of the work that we publish. This form provides structure for consistency and transparency in reporting. For further information on Nature Portfolio policies, see our Editorial Policies and the Editorial Policy Checklist.

## Statistics

For all statistical analyses, confirm that the following items are present in the figure legend, table legend, main text, or Methods section.

| n/a | Confirmed | |
|---|---|---|
| ☐ | ☒ | The exact sample size ($n$) for each experimental group/condition, given as a discrete number and unit of measurement |
| ☐ | ☒ | A statement on whether measurements were taken from distinct samples or whether the same sample was measured repeatedly |
| ☐ | ☒ | The statistical test(s) used AND whether they are one- or two-sided<br>*Only common tests should be described solely by name; describe more complex techniques in the Methods section.* |
| ☐ | ☒ | A description of all covariates tested |
| ☐ | ☒ | A description of any assumptions or corrections, such as tests of normality and adjustment for multiple comparisons |
| ☐ | ☒ | A full description of the statistical parameters including central tendency (e.g. means) or other basic estimates (e.g. regression coefficient) AND variation (e.g. standard deviation) or associated estimates of uncertainty (e.g. confidence intervals) |
| ☒ | ☐ | For null hypothesis testing, the test statistic (e.g. $F$, $t$, $r$) with confidence intervals, effect sizes, degrees of freedom and $P$ value noted<br>*Give P values as exact values whenever suitable.* |
| ☐ | ☒ | For Bayesian analysis, information on the choice of priors and Markov chain Monte Carlo settings |
| ☐ | ☒ | For hierarchical and complex designs, identification of the appropriate level for tests and full reporting of outcomes |
| ☐ | ☒ | Estimates of effect sizes (e.g. Cohen's $d$, Pearson's $r$), indicating how they were calculated |

*Our web collection on statistics for biologists contains articles on many of the points above.*

## Software and code

Policy information about availability of computer code

| | |
|---|---|
| Data collection | Data was collected through an online experiment executed with Dallinger v5.1.0. |
| Data analysis | Analyses were conducted in JAGS (v4.3.2) using the R (v4.4.3) package rjags (v4-17). Data and analysis code is available at https://osf.io/7yrts/. |

For manuscripts utilizing custom algorithms or software that are central to the research but not yet described in published literature, software must be made available to editors and reviewers. We strongly encourage code deposition in a community repository (e.g. GitHub). See the Nature Portfolio guidelines for submitting code & software for further information.

## Data

Policy information about availability of data

All manuscripts must include a data availability statement. This statement should provide the following information, where applicable:

- Accession codes, unique identifiers, or web links for publicly available datasets
- A description of any restrictions on data availability
- For clinical datasets or third party data, please ensure that the statement adheres to our policy

The cultural evolutionary model and gene-culture coevolutionary model code is provided. The output of the models is shown in the figures and discussed in the results, but was not saved. Rather the models themselves are distributed in place of any specific output.

For the experiment, the raw data (per-trial, per-participant) is provided as a text file that an be loaded as a dataframe in R.

All datasets were generated by the authors during the course of the study and contain no identifying or sensitive information.

## Research involving human participants, their data, or biological material

Policy information about studies with human participants or human data. See also policy information about sex, gender (identity/presentation), and sexual orientation and race, ethnicity and racism.

| | |
|---|---|
| Reporting on sex and gender | n/a<br><br>Sex and gender were not relevant to our study questions. As such we did not restrict participation by sex or gender or ask participants to provide this information. Neither did we include sex or gender as a factor in any analyses.<br><br>For general characteristics of the population we recruited from, see below. |
| Reporting on race, ethnicity, or other socially relevant groupings | n/a |
| Population characteristics | Participants were recruited through Amazon's Mechanical Turk. While we did not collect demographic data, several studies have examined the demographics of Mechanical Turk workers and provide relevant information. The total worker population is estimated to be stable at over 100 000 people, although the effective population size may be smaller, with around 2000 workers active at any one time. Most workers are US-based, with a sizable number of Indian workers, and the US and Indian sub-populations being most active at different times of day. This may explain why participant characteristics and behavior can also vary by time of day, as well as on weekends versus weekdays. The worker population is diverse and somewhat comparable to the overall US population in terms of age, sex, education and socio-economic status, and is significantly more diverse than typical undergraduate populations. Nonetheless, it is younger, more female, better educated and less rich than the US population as a whole.<br><br>As sex, gender and age were not relevant to our study we did not filter by these variables or record them for our participants. Mechanical Turk automatically limits participation to adults (i.e. those 18 years and older). |
| Recruitment | Participants were recruited through Amazon's Mechanical Turk. See above for demographic factors. Other than Mechanical Turk's default filtering, which excludes workers under 18 years old and those who reliably fail to complete tasks, no other filtering was applied.<br><br>Participants were paid $3 for completing the experiment, plus a bonus payment for each correct judgement made (including both initial and final judgements), totaling $3 if all trials were answered correctly. |
| Ethics oversight | Ethical approval was granted by the Arizona State University IRB (Study ID: 00004815).<br><br>Prior to beginning the study, participants were briefed on its design and their informed consent was obtained. |

Note that full information on the approval of the study protocol must also be provided in the manuscript.

# Field-specific reporting

Please select the one below that is the best fit for your research. If you are not sure, read the appropriate sections before making your selection.

☐ Life sciences ☒ Behavioural & social sciences ☐ Ecological, evolutionary & environmental sciences

For a reference copy of the document with all sections, see nature.com/documents/nr-reporting-summary-flat.pdf

# Behavioural & social sciences study design

All studies must disclose on these points even when the disclosure is negative.

| | |
|---|---|
| Study description | Study was an online experiment, data are quantitative. The study was approved by Arizona State University IRB (Study ID: 00004815). |
| Research sample | Participants and demographics<br>Participants were recruited through Amazon Mechanical Turk (MTurk). We did not collect demographic variables such as age or sex. However, prior large-scale characterizations of the MTurk workforce indicate that it is more diverse than typical undergraduate samples, while being younger, more female, better educated, and less wealthy than the general U.S. population. The platform includes substantial U.S. and Indian user bases, with participation varying by time of day. Because demographic data were not collected, the representativeness of the sample cannot be directly assessed, but it is likely comparable to standard MTurk participant pools used in behavioural research.<br><br>Beyond these biases in the demographic composition of MTurk populations there are likely no other biases. We do not anticipate these biases had any systematic effect on the results of this study.<br><br>Rationale for using MTurk |

| | |
|---|---|
| | MTurk was chosen because it allows rapid recruitment of a large number of independent participants for real-time, group-based online tasks, which was required for our experimental design. |
| Sampling strategy | Sampling procedure<br>A convenience sampling approach was used. The study was posted on MTurk and participants self-selected into the task on a first-come, first-served basis until the target sample size was reached. Participants were assigned to groups in the order they arrived.<br><br>Sample size and rationale<br>A total of 800 participants were recruited and arranged into 80 groups of 10, with 20 groups assigned to each of the four experimental conditions. No formal statistical power analysis was used to predetermine sample size. Instead, we selected the largest sample that was practical to recruit and compensate within project resource constraints. The resulting dataset provides clear and stable estimates of the primary effects of interest, indicating that the sample size was sufficient for the study's aims. |
| Data collection | Data was recorded automatically by the experimental software, Dallinger. Participants completed the study remotely and so the experimenter was not present. |
| Timing | Data were collected between October 1st and October 7th, 2019. |
| Data exclusions | Participants were excluded from the analyses if they failed to complete the study, i.e. if they did not fill out the debriefing or if they did not answer any trials. This removed 134 of 800 participants, leaving 666.<br><br>In addition, in the first analysis, participant responses were excluded on trials where fewer than five participants remained in their group. This removed 2,611 responses, including all responses made by 28 participants, leaving 20,035 decisions made by 638 participants in the analysis.<br><br>In the second analysis, participants were excluded if they did not complete all 50 trials or if fewer than five participants in their group completed all fifty trials. This removed 214 of 666 participants, with 452 participants remaining in the analysis. |
| Non-participation | Of the initially recruited 800 participants, 134 withdrew without completing the experiment. As they were recruited through Mechanical Turk no reason for withdrawal was required. As participants completed the study in groups of 10, and had to wait for all group members to answer each question before moving to the next, it is likely that participants who left did so because they found waiting for their group to fill or waiting for their group mates to respond to be excessively slow. |
| Randomization | Participants were allocated to groups at random, and groups were assigned to the experimental conditions at random. |

# Reporting for specific materials, systems and methods

We require information from authors about some types of materials, experimental systems and methods used in many studies. Here, indicate whether each material, system or method listed is relevant to your study. If you are not sure if a list item applies to your research, read the appropriate section before selecting a response.

## Materials & experimental systems

| n/a | Involved in the study | |
|---|---|---|
| ☒ | ☐ | Antibodies |
| ☒ | ☐ | Eukaryotic cell lines |
| ☒ | ☐ | Palaeontology and archaeology |
| ☒ | ☐ | Animals and other organisms |
| ☒ | ☐ | Clinical data |
| ☒ | ☐ | Dual use research of concern |
| ☒ | ☐ | Plants |

## Methods

| n/a | Involved in the study | |
|---|---|---|
| ☒ | ☐ | ChIP-seq |
| ☒ | ☐ | Flow cytometry |
| ☒ | ☐ | MRI-based neuroimaging |

## Plants

| | |
|---|---|
| Seed stocks | n/a |
| Novel plant genotypes | n/a |
| Authentication | n/a |
